# Microarray-Based Gene Expression Profiling to Elucidate Effectiveness of Fermented *Codonopsis lanceolata* in Mice

**DOI:** 10.3390/ijms15045907

**Published:** 2014-04-08

**Authors:** Woon Yong Choi, Ji Seon Kim, Sung Jin Park, Choong Je Ma, Hyeon Yong Lee

**Affiliations:** 1 Department of Medical Biomaterials Engineering, Kangwon National University, Chuncheon 200-701, Korea; E-Mails: cwy1012@hanmail.net (W.Y.C.); kimjiseon@kangwon.ac.kr (J.S.K.); cjma@kangwon.ac.kr (C.J.M.); 2 Department of Tourism Food Service Cuisine, Hallym Polytechnic University, Gangwon 200-711, Korea; E-Mail: psjmsj@hanmail.net; 3 Department of Food Science and Technology, Seowon University, Cheongju, Chungbuk 361-742, Korea

**Keywords:** fermented *Codonopsis lanceolata*, microarray, gene expression, obesity

## Abstract

In this study, the effect of *Codonopsis lanceolata* fermented by lactic acid on controlling gene expression levels related to obesity was observed in an oligonucleotide chip microarray. Among 8170 genes, 393 genes were up regulated and 760 genes were down regulated in feeding the fermented *C. lanceolata* (FCL). Another 374 genes were up regulated and 527 genes down regulated without feeding the sample. The genes were not affected by the FCL sample. It was interesting that among those genes, Chytochrome P450, Dmbt1, LOC76487, and thyroid hormones, *etc.*, were mostly up or down regulated. These genes are more related to lipid synthesis. We could conclude that the FCL possibly controlled the gene expression levels related to lipid synthesis, which resulted in reducing obesity. However, more detailed protein expression experiments should be carried out.

## 1. Introduction

The worldwide incidence of obesity continues to escalate despite increased awareness and global efforts to identify its origins. In essence, down regulated energy homeostasis stems from a reduction in physical activity and an increase in the accessibility of the energy-dense foods, combined with a myriad of genetic, social, and economic factors [1]. Abdominal obesity often results in a clustering of atherogenic risk factors including hypertension, dyslipidemia, alterations in imflammatory cytokine profiles, and hyperinsulinemic insulin resistance, all of which lead to an increase in cardiovascular disease [2]. Accordingly, many studies have been conducted to delineate the relationship between increased adiposity and insulin resistance. The results of these studies have identified adipokines as potential factors involved in insulin resistance [3,4].

Some phytochmicals in traditional medicinal plants have hypolipidaemic and antioxidative effects, modulating gene expression in lipid and lipoprotein metabolism-related genes such as low density lipoprotein (LDL) receptors (LDL recptors), 3-hydroxy-3-methyglutaryl-coenzyme A (HMG-CoA) reductase, *etc*., and antioxidant defense-related genes such as glucose-6-phosphate dehydrogenase (G6PD) and glutathione S-transferase (GST), *etc*. To understand the correlation between gene expression and cell metabolisms, especially for pharmacogenomics, micro-array analysis has been widely used [5]. For example, novel determinants of response to chemotherapy in colon cancer were identified by using pharmacogenomic approach [6,7]. The roots of *Codonopsis lanceolata* (CL) have been used as a traditional medicinal herb to treat several inflammatory diseases, memory impairment and inhibitory action of obesity in far east regions [8,9]. Some of them were obtained as they were (fresh or dry type) or simply extracted by hot water. However, their tastes were not favorable due to different flavors and colors of saponins and its active components were not well eluted during conventional extraction processes [10]. Nonetheless, they contain various active components including tannins, saponins, polyphenolics, alkaloids, essential oils and steroids [11].

To overcome this kind of limitation in utilizing natural plants, the lactic acid fermentation process has often been employed to easily elute active components and to improve transportation into the cells by the help of microorganisms [10]. For making better utilization of *C. lanceolata*, one of the edible lactic acids was fermented with the herb, and the fermented extracts were tested by a cDNA microarray method to monitor approximately 8170 gene expression changes in the liver of mice.

## 2. Results and Discussion

**Figure 1.**
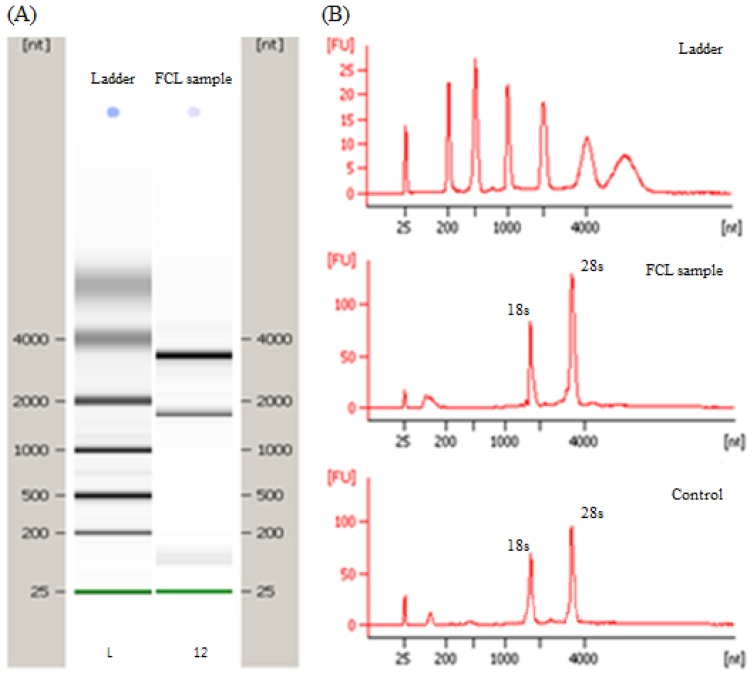
Quality of total RNA isolated for microarray. The total RNA was prepared from liver tissue of mice. Image of gel electrophoresis was photographed (**A**) and the quality of RNA was calculated from scanned image (**B**).

Figure 1 shows the quality and quantity of total RNAs extracted from mice liver fed by FCL sample and also the control, by comparing with the standards. It was shown that the sample RNAs extracted from both samples showed clear bands and also sharp two peaks of 18s and 28s in the scanned image, which confirmed that the RNAs from both samples were good enough to perform microarray experiment. The whole genome variation of mice liver was observed by using Agilent mice whole genome 4 × 44K oligomicroarray. They mostly showed red or green spots on the glass, but some had a yellow color because most genes were changed significantly after feeding FCL (data not shown) in this case.

**Figure 2.**
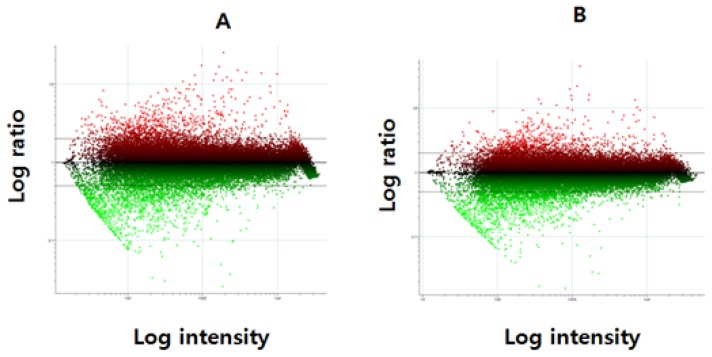
Normalization of microarray (MA plot). Primary data from raw image of control group only fed with the commercial feeds (**A**) and the fermented *C. lanceolata* (FCL) group fed with the fermented sample (**B**) were normalized using the lowess method.

These raw images were normalized by MA plot using locally weighted scatterplot smoothing (LOWESS) method in Figure 2. We applied the normalization process. A skewed form of ratio pattern before normalization changed to a linear pattern centered to zero after normalization, which implies that specific genes, *i.e.*, not all of the genes related to obesity, were noticeably changed after feeding the FCL samples. To understand which gene expression was changed, hierarchical clustering analysis was performed by using 8170 genes.

**Figure 3.**
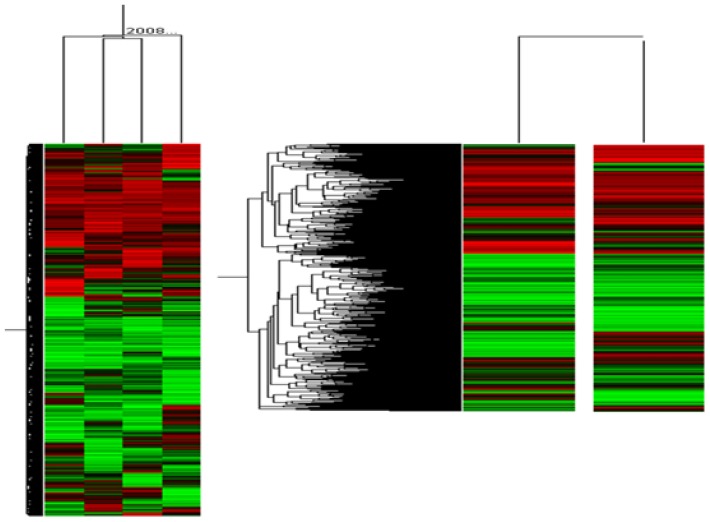
Hierarchical clustering analysis of genes changed expression in control group only fed with the commercial feeds and the FCL group fed with the fermented sample. The genes are up- or down-regulated more 2.0-fold in FCL-treat mice. Relative gene expression is measured as the ratio of the two fluorescences: “up-regulation” of the experimental transcription relative to the control visualized in red, “down regulation” show in green.

As a result, as shown in Figure 3, 1153 genes were up or down regulated two times higher than the case of feeding the control. Specifically, up-regulation of 393 genes (red color cluster) and down-regulation of 760 genes (green color in cluster) was observed. In addition, it should be noted that a further 374 genes were up regulated and 527 genes down regulated in feeding the commercial diets as the control. However there was no change in those fed the FCL, which were not strongly related to lipid synthesis. At this time, Signal-to-Noise Ratio (SNR) has not been considered even though some background DNA could affect the results of the cluster analysis. This is because, in this work, not many DNA samples were used and we were more interested in what kinds of genes could be mostly regulated at first. However, for more exact expectation of gene expression, SNR thresholds will be evaluated for further work [12].

Among them shown in Figure 3, the names of most significantly regulated genes were listed in Table 1, in feeding the FCL. It was interesting that Chytochrome P450, Dmbt1, LOC76487, and thyroid hormones were mostly regulated, which are more related with general metabolic processes in the liver. This result implies that the FCL could play a more important role in controlling lipid synthesis, which could result in controlling the obesity, as reported in other works [12–14]. However, to clearly understand this controlling mechanism, further gene annotation analysis using protein interaction database should be identified as well as the regulation patterns of more obesity related genes.

## 3. Experimental Section

### 3.1. Sample Preparation

The roots of *Codonopsis lanceolata* used for this experiment were purchased from Hoengseong (Gangwon, Korea). Then the roots were washed and dried. The dried roots were ground by a mixer before adding into a 1 L fermentor jar with 5% (*w*/*v*) of *Leuconostoc mesenterocides* (KCCM 35471) inoclum. The fermentation process was carried out in two steps. First, 7 days after inoculation, the sample was fermented at 30 °C and 75% of humidity, and then they were fermented again at 20 °C for 7 more days. Then, the culture broth was filtered by a membrane filter to obtain solid parts from the culture medium. The solid part, fermented *C. lanceolata* was extracted using reflux for 24 h with 1:10 volume of water, and the extracts were concentrated by a rotary vacuum evaporator (CCA-1100, Eyela, Tokyo, Japan). The sample was freeze-dried and called as fermented *C. lanceolata* (FCL). The extraction yield of the fermented *C. lanceolata* was as 38.4% (*w*/*w*), and the extract was dissolved in distilled water immediately before oral administration.

### 3.2. Animals and Feeding Protocols

Nine-week-old male ICR mice (Sprague Dawley, Oriental Co., Ltd., Seoul, Korea) were used with a commercial pellet diet and water *ad libitum* for 7 days of acclimatization as follows: Five mice were housed in groups of six in stainless steel cages in a room (total 30 mice) maintained at under a constant temperature (23 ± 1 °C) and humidity (60% ± 10%) under a 12-h light/dark cycle (light on am 07:30–pm 07:30). Just before starting the experiment, the prescription was distilled water, and then mice were orally fed with the FCL at the dosage of 1 mL/g body weight for 21 days. As a control group, the same 30 mice were fed only with the same diet except for the FCL under the same conditions. After the last bleed at 3 weeks, all of the mice from both groups were killed and their livers were removed, rinsed with phosphate-buffer saline (PBS). A cross section of the left lateral lobe of the liver was collected in 10% formalin for histological analysis and the remaining liver tissue was collected in liquid nitrogen [13]. The liver sample was stored at −80 °C until it was analyzed.

### 3.3. RNA Extraction and Purification

Isolated liver was homogenized and total RNA was extracted by using Trizol reagent (Invitrogen, Carlsbad, CA, USA) according to the manufacturer’s instruction for the determining mRNA expression levels. 100 μg of RNA from each sample were purified by using the RNeasy RNA purification column (Qiagen, Hilden, Germany) following the producer’s protocol. Residual DNA was removed by an additional on-column DNase digestion step using the Quagen RNase-free DNase set [15].

### 3.4. Oligonucleotide Microarray Assay

Exactly 100 pmole/μL of oligonucleotide probes were printed on ultra GAPS glass slides (Corning Life Science, New York, NY, USA) using a PixSys robotic printer (Cartesian Technologies, Inc., Irvine, CA, USA) with four duplicates for each probe, where each slide contained four arrays, each with 44,000 spots by using an Agilent 4 × 44K two-color microarray (G4846A, Technologies Inc., Santa Clara, CA, USA). Then, the probe was fixed by US cross linking followed by the manufacturer’s protocol (Corning Life Science, New York, NY, USA) [14]. For control and test RNAs, the synthesis of target cRNA probes and hybridization were performed using Agilent’s Low RNA Input Linear Amplification kit (Agilent Technology, Waldbronn, Germany) according to the manufacturer’s instructions. Briefly, each 5 μg of total RNA from the control and FCL groups, respectively and T7 promoter primer were mixed and incubated at 65 °C for 10 min. cDNA master mix (5× First strand buffer, 0.1 M DTT, 10 mM dNTP mix, RNase-Out, and MMLV-RT) was prepared and added to the reaction mixer. The samples were incubated at 40 °C for 2 h and then the RT and dsDNA synthesis was terminated by incubating at 65 °C for 15 min. The reverse-transcription master mix was prepared as the manufacturer’s protocol (4× Transcription buffer, 0.1 M DTT, NTP mix, 50% PEG, RNase-Out, Inorganic pyrophosphatase, T7-RNA polymerase, and Cyanine 3/5-CTP). Transcription of dsDNA was performed by adding the transcription master mix to the dsDNA reaction samples and incubating at 40 °C for 2 h. Amplified and labeled cRNA was purified on cRNA Cleanup Module (Agilent Technology, Waldbronn, Germany) according to the manufacturer’s protocol. Labeled cRNA target was quantified using ND-1000 spectrophotometer (NanoDrop Technologies, Inc., Wilmington, DE, USA) [13]. After checking labeling efficiency, fragmentation of cRNA was performed by adding 10× blocking agent and 25× fragmentation buffer and incubating at 60 °C for 30 min. The fragmented cRNA was resuspended with 2× hybridization buffer and directly pipetted onto assembled Agilent’s Whole Rat Genome Oligo Microarray (44K). The arrays hybridized at 65 °C for 17 h using Agilent Hybridization oven (Agilent Technology, Waldbronn, Germany).

### 3.5. Microarray Data Analysis

The hybridized images were scanned using Agilent’s DNA microarray scanner and quantified with Feature Extraction Software (Agilent Technology, Palo Alto, CA, USA). All data normalization and selection of fold-changed genes were performed using GeneSpringGX 7.3 (Agilent Technology, Waldbronn, Germany). Intensity-dependent normalization locally weighted scatterplot smoothing (LOWESS) was performed, where the ratio was reduced to the residual of the Lowess fit of the intensity *vs.* ratio curve. The averages of normalized ratios were calculated by dividing the average of normalized signal channel intensity by the average of normalized control channel intensity. Functional annotation of genes was performed according to The Gene Ontology Consortium [16] by GeneSpringGX 7.3. Gene classification was based on searches done by BioCarta [17], GenMAPP [18], DAVID [19], and Medline databases [5,20].

### 3.6. Statistical Analysis

The data are expressed as mean ± SD (standard deviation) and are the average values of three to five values per experiment. The data were analyzed by using the SPSS package (Version 10.0, SPSS, Chicago, IL, USA). Analysis of variance (ANOVA) was conducted, and Ducan’s multiple range tests were used to determine the significance of differences between groups. The level of statistical significance was set to *p* < 0.01.

## 4. Conclusions

It was first reported here that the extract from the fermentation of *Codonopsis lanceolata*, most widely used medicinal herb in Asia, could play a role in reducing the obesity by controlling the gene expression levels related to the obesity. Three hundred and ninety three genes out of 8170 genes were up regulated, while 760 genes were down regulated. Among those genes, ChytochromeP450, Dmbt1, LOC76487, and thyroid hormones, *etc.*, which are more closely related to lipid synthesis, were mostly up or down regulated. It was also interesting that, for the control group as a placebo, about 374 and 727 genes were up and down regulated, respectively. These genes are not strongly related to lipid synthesis. Therefore, these results could imply that the fermented sample reduced/controlled the obesity better than that from conventional water extraction such as 38.4% *vs.* 23.58%, respectively (data not shown). However, more in-depth studies on both profiling the gene regulation directly related to obesity and identifying active substances through the lactic acid fermentation process should be carried out and should be associated with real-time PCR analysis.

## Figures and Tables

**Table 1.** The list of significantly up or down regulated genes in mouse liver treated with fermented *Codonopsis lanceolata*.

Up-regulated gene (>2.0-fold)		Down-regulated gene (<0.5-fold)	
Name	Fold	Name	Fold
Cytochrome P450, family 4, subfamily a. polypeptide 14 (Cyp4a14)	14.28 ± 0.39	Thyroid hormone responsive SPOT14 homolog (Rattus)	0.11 ± 0.40
Deleted in malignant brain tumors 1 (Dmbt1)	12.47 ± 0.13	Fatty acid syhthase	0.18 ± 0.27
Homolog to homo sapiens Cytochrome P450 4A11 precursor	10.24 ± 0.90	Transcribed locus	0.21 ± 0.26
PREDICTED: hypothetical protein LOC76487 [Mus musculus]	8.74 ± 0.72	Farnesyl diphosphate synthetase (Fdps)	0.22 ± 0.49
Metallothionein 2	8.42 ± 0.50	Mid1 interacting protein 1 (gastrulation specific G12-like (zebrafish))	0.24 ± 0.44
Deleted in malignant brain tumors 1	8.01 ± 0.45	Sterol regulatory element binding protein 1 (Srebp1)	0.24 ± 0.39
Cytochrome P450, family 4, subfamily a. polypeptide 10	7.91 ± 0.44	NDA(P) dependent steroid dehydrog	0.25 ± 1.47
Metallothionein 1	7.41 ± 0.96	Transmembrane inner ear (Tmie)	0.28 ± 0.84
RIKEN cDNA A930041I02 gene (A930041I02Rik)	7.12 ± 0.50	Amino carboxymuconate semialdehyde decarboxylase (Acmsd)	0.31 ± 0.47
Metallothionein 2	6.82 ± 0.28		
